# Children bereaved by fatal intimate partner violence: A population-based study into demographics, family characteristics and homicide exposure

**DOI:** 10.1371/journal.pone.0183466

**Published:** 2017-10-04

**Authors:** Eva Alisic, Arend Groot, Hanneke Snetselaar, Tielke Stroeken, Elise van de Putte

**Affiliations:** 1 Monash University Accident Research Centre, Monash University, Melbourne, Australia; 2 Psychotrauma Centre Wilhelmina Children’s Hospital, University Medical Centre Utrecht, Utrecht, the Netherlands; 3 Wilhelmina Children’s Hospital, University Medical Centre Utrecht, Utrecht, the Netherlands; Tilburg University, NETHERLANDS

## Abstract

**Background:**

In the context of violence against women, intimate partner homicide increasingly receives research and policy attention. Although the impact of losing a parent due to intimate partner homicide is intuitively obvious, little is known about the children involved. We aimed to identify all children bereaved by parental intimate partner homicide in the Netherlands in the period 2003–2012, describe their demographics and family circumstances, and assess their exposure to prior violence at home and to the homicide itself.

**Methods and findings:**

We cross-examined 8 national data sources and extracted data about children’s demographics and circumstances prior to, and during the homicide. Our primary outcomes were prior violence at home (child maltreatment, neglect or domestic violence) and homicide witness status (ranging from being at a different location altogether to being present at the scene). During the decade under study, 256 children lost a biological parent due to 137 cases of intimate partner homicide. On average, the children were 7.4 years old at the time of the homicide (51.1% were boys; 95% CI 47.3–54.7) and most lost their mother (87.1%; full population data). Immigrant children were overrepresented (59.4%; 95% CI 52.8–66.0). Of the children for whom information about previous violence at home was gathered, 67.7% (95% CI 59.7–73.7) were certainly exposed and 16.7% (95% CI 11.3–22.2) probably. Of the children who had certainly been exposed, 43.1% (95% CI 41.1–60.9) had not received social services or mental health care. The majority of the children (58.7%; 95% CI 52.1–65.3) were present at the location of the homicide when the killing took place, with varying levels of exposure. Homicide weapons mostly involved cutting weapons and firearms, leading to graphic crime scenes.

**Conclusions:**

Care providers need capacity not only to help children cope with the sudden loss of a parent but also with unaddressed histories of domestic violence and exposure to graphic homicide scenes, in a culture-sensitive way. Future directions include longitudinal monitoring of children’s mental health outcomes and replication in other countries.

## Introduction

Intimate partner violence has gained widespread and high-level attention in the past few years, reflected in e.g. the United Nations (UN) Commission on the Status of Women, the UN Secretary-General’s UNiTE Campaign, the World Health Organization’s priorities, and the 2030 Sustainable Development Agenda [1–4]. Its fatal extreme, intimate partner homicide, is increasingly considered in research and policy efforts [3–5], fueled by the finding that 39% of homicides of women worldwide are due to intimate partner violence, compared to 6% of male homicides [6].

Many victims of intimate partner homicide are parents [7]. However, little is known about the bereaved children’s circumstances and support needs. A recent systematic review identified only 13 broadly relevant studies worldwide, of which 8 were case studies, and all but two were convenience samples [8]. Possible reasons for the lack of research and insight include the highly sensitive nature of the topic, repeated changes in children’s living arrangements and contact details after the homicide, dissatisfaction of caregivers with child protection and placement services, and high levels of grief and mental health problems among both children and caregivers, including avoidance of potential reminders.

Clinical case series describe substantial mental health and development difficulties in children exposed to fatal domestic violence. Many mental health difficulties reside within the domains of Posttraumatic Stress Disorder (PTSD) and traumatic grief, and include intrusive memories, anxiety, sleep disturbances, aggressive and self-destructive behavior, protracted grief, hyperactivity, and concentration problems [9–15]. Prominent developmental difficulties involve attachment problems in relation to new caregivers, regression (e.g. language deterioration), social problems, identity questions, and deteriorating school performance. In the long term, there are concerns about the heightened risks for children of becoming a perpetrator of violence [10].

Two factors that appear to compound the burden of the traumatic parental loss and have implications for services and treatment include a) being a witness to the killing and b) exposure to previous violence at home. In the clinical data available, children who witnessed the homicide had higher levels of PTSD symptomatology, more persistent emotional and behavioral problems, and more difficulty to go through the grieving process than children who did not witness the killing [12, 15]. Similarly, children who have been exposed to violence prior to the homicide are thought to be at particular risk. The broader child abuse literature suggests that previous exposure to neglect, maltreatment, and partner violence at home consists a risk factor for both mental health problems and violence perpetration later in life [16–19].

It is virtually unknown what the demographic and family characteristics of the population of children bereaved by intimate partner homicide are, and to which extent these children have been exposed to the killing itself and previous violence. In 2004, Lewandowski and colleagues published a pioneering study in the USA that included 73 cases of femicide affecting 146 children, 125 or 126 of them biological children of the victim (the remaining 20–21 were stepchildren, other relatives, or nonrelatives living in the same household) [7]. The study’s results were disturbing. In two thirds of the homes, there had been prior physical assault of the mother. In 35% of the families, at least one child witnessed the murder. In another 37% of the families, a child found the victim’s body. These findings are in urgent need of replication and extension. First, the situation may be different in other regions or countries (e.g. due to differences in socio-economic, legal and health care characteristics). Second, because this study made use of proxy reports, it was dependent on tracing potential informants and obtaining their consent to participate. For approximately 40% of the femicides, an appropriate and consenting informant was found [20]; it is unclear whether these cases are representative of the population of children affected, and how well the informants were able to assess children’s situations.

In the current study, we aimed to identify all children exposed to parental intimate partner homicide in the Netherlands in the decade 2003–2012 (while they were 0–18 years old), and describe their characteristics and circumstances prior to, and during the homicide. The study is part of a larger Dutch project on the consequences of fatal domestic violence, covering the period 1993–2012 [21]. Because preliminary enquiries indicated that databases covering the first decade were only partially digitalized and not available for comprehensive searches, we focused on the second decade (2003–2012). Our research questions were the following:

1. What are the demographic and family characteristics of children bereaved by intimate partner homicide?
2. To what extent have children been exposed to previous violence at home, including neglect, child maltreatment, and partner violence?
3. To what extent have children been exposed to the killing?

The answers to these questions provide potentially important input for planning adequate trauma-informed care (i.e. care that builds on awareness of the impact of psychological trauma and tailors services accordingly [22]).

## Materials and methods

The protocol for the larger project has been published [21] and has been approved by the University Medical Center Utrecht Ethics Committee (13/609, 24-12-2013). For the current article, we aimed to systematically study all children who had been bereaved by parental intimate partner homicide in the Netherlands over a period of 10 years (2003–2012). We considered all cases in which a biological parent was killed due to intimate partner violence, either by a current or a former partner. Due to their sensitive nature, the data can only be shared after approval by the ethics committee.

We examined eight sources of data, chosen based on their likelihood of documenting intimate partner homicide and/or the circumstances of children bereaved by intimate partner homicide, as well as accessibility. The eight sources included a) databases of the Child Care and Protection Board, b) databases of the national Youth Care Agency, c) databases of the Department of Justice, d) two criminology books that describe homicide cases in the Netherlands [23],[24], and their accompanying data; e) the ‘Elsevier Murderlists’; brief descriptions of homicides published in the national magazine Elsevier; f) the client database of the National Psychotrauma Centre, based at the Wilhelmina Children’s Hospital; g) the public database of legal verdicts (www.rechtbank.nl); and h) the database Lexis Nexis that includes articles of all national and regional newspapers in the Netherlands.

All data sources involved written information. Some, such as the Elsevier Murderlists and the database of legal verdicts, were open to the public. For all non-public databases, we obtained access via the relevant authorities or owners, with permission from the ethics committee. Cases mentioned in newspapers had to be confirmed by at least one other source. We excluded cases in which the victim and/or perpetrator were not Dutch residents, as well as cases in which the verdict was ‘not guilty’ or ‘involuntary manslaughter’.

We extracted the following information regarding children’s background and circumstances: a) demographic characteristics of the perpetrator, victim and child(ren) (age, gender, ethnicity); b) characteristics of the family (previous partner violence, child maltreatment or child neglect, relationship status and living arrangement of victim and perpetrator, previous involvement of social or mental health care services); and c) characteristics of the homicide (weapon, witness/victim status of the children, location). None of the sources provided complete data (e.g. the books provided limited data regarding previous violence). Moreover, the information was often not recorded in a standardized manner; information was prepared by people with varying professions for varying purposes at various points in time. For example, for some children we obtained one brief letter about a child protection decision two weeks after the homicide, while for other children we had extensive legal and social information that had been put together over a period of five years since the killing.

Almost all information was in narrative form and had to be categorized by the research team [21]. For this purpose, we developed a coding scheme within IBM SPSS Statistics version 22.0, which was tested with two cases, discussed within the team and with the study’s advisory board, adjusted, tested with two further cases, and subsequently deployed for data extraction. The research team had continuous (weekly) meetings in which coding and coding decisions were discussed. The main codes are the ones shown in the tables in the results section. We collected case files in 2014 and 2015, and extracted and analyzed the data in 2015 and 2016.

For the analyses, we used descriptive statistics, mainly involving proportions with 95% confidence intervals (when needed, these were adjusted in line with the full population data; see Table 1) and averages with standard deviations. In the text of the results section, we described proportions based on the number of cases for which information was available; the tables also show percentages based on the total number of cases. We compared information about immigration status with national population data [25] using Chi-square tests. All analyses were conducted in IBM SPSS Statistics version 22.0.

**Table 1:** Characteristics of children bereaved by intimate partner homicide in the Netherlands, 2003–2012 (N = 256).

	%	Valid %	95% confidence interval for valid %^a^
Gender (N = 237)			
Boys	47.3	51.1	47.3–54.7
Girls	45.3	48.9	45.3–52.7
Unknown	7.4		
Age at time of homicide (N = 213)			
Younger than 10 years	55.5	66.7	60.3–72.3
10 years and older	27.7	33.3	27.7–39.7
Unknown	16.8		
Ethnicity (N = 212)			
Autochthonous	33.6	40.6	34.0–47.2
Allochthonous	49.2	59.4	52.8–66.0
Unknown	17.2		
Guardian/custodian (N = 189)			
Both biological parents	50.8	68.8	62.2–75.4
Mother	20.3	27.5	21.1–33.9
Father	1.2	1.6	1.2–3.4
Agency	0.8	1.1	0.8–2.5
Other (e.g. in network)	0.8	1,1	0.8–2.5
Unknown	26.2		
Living with whom at the time of the homicide (N = 223)			
Biological parents	40.2	46.2	40.2–52.7
Mother	32.0	36.8	32.0–43.1
Mother with new partner	6.3	7.2	6.3–10.6
Father	2.0	2,2	2.0–4.2
Father with new partner	2.0	2.2	2.0–4.2
Elsewhere	4.7	5.4	4.7–8.3
Unknown Previous exposure to domestic violence/maltreatment/neglect (N = 174)	12.9		
Certainly yes^b^	45.3	66.7	59.7–73.7
Probably yes	11.3	16.7	11.3–22.2
Probably no	11.3	16.7	11.3–22.2
Unknown	32.0		

^a^ 95% CI’s were adjusted when a boundary was impossible (e.g. the statistical upper boundary for boys was 57.4% while we knew that at least 45.3% of the population was female, hence the truly possible upper boundary for boys was 54.7%).

^b^ 43.1% (95% CI 41.1–60.9) of these children had not received social services or mental health care.

## Results

### Demographics and family characteristics

Over a period of 10 years, 256 children lost a parent due to intimate partner homicide (see Table 1). The total number of cases was 137 (see Table 2). On average, the children were 7.4 years old at the time of the homicide (SD = 4.9, Median = 7.0, range 0–17 years). About two thirds of them were younger than 10 years old. Well over half of the children were considered immigrants (‘allochthonous’; they or at least one of their parents were/was born outside of the Netherlands), which is high compared to the proportion of immigrants in the general population (21% in 2014 [25]; Chi-square for *N* = 212 was 188.764, with p < .001). This overrepresentation could not be explained by larger family size for immigrant families; the rate of immigrants among the victims was similarly high (52.3%). Most children were living with both biological parents or with their mother at the time of the homicide. The families had 1 to 5 children under the age of 18 years (Mean = 1.88; SD = .97), and 1 to 6 children of any age (Mean = 2.13; SD = 1.08).

**Table 2:** Characteristics of the victim-perpetrator couples (N = 137).

	%	Valid %	95% confidence interval for valid %^a^
Victim (N = 137 cases)			
Mother	87.6	87.6	n/a
Father	12.4	12.4	n/a
Unknown	0.0		
Perpetrator (N = 137 cases)			
The other biological parent (87.5% male)	64.2	64.2	64.2–67.8
(Former) partner (not biological parent; 84.6%)	32.1	32.1	32.1–38.7
Unclear whether biological parent (100% male partner)	3.6	3.6	
Unknown			
Ethnicity (N = 99 cases for which both were known)			
Dutch-Dutch	32.1	44.4	34.7–54.2
Turkish-Turkish	7.3	10.1	7.3–16.0
Moroccan = Moroccan	6.6	9.1	6.6–14.8
Surinam-Surinam	3.6	5.1	3.6–9.4
Other^b^	22.6	31.3	22.6–40.4
Unknown	27.7		
Number of children per victim^c^			
Minors only (<18 years old; N = 137 cases)			
1	43.1	43.1	n/a
2	35.0	35.0	n/a
3	14.6	14.6	n/a
4	5.8	5.8	n/a
5	1.5	1.5	n/a
Unknown	0.0		
All children (incl >18 years old; N = 133 cases)			
1	31.4	32.3	31.4–34.3
2	36.5	37.6	36.5–39.4
3	19.0	19.5	19.0–21.9
4	6.6	6.8	6.6–9.5
5	2.9	3.0	2.9–5.8
6	0.7	0.8	0.7–2.2
Unknown	2.9		
Relationship status at the time of the homicide			
Living arrangement (N = 122 cases)			
Living together	48.2	54.1	48.2–59.1
Separated	38.0	42.6	38.0–59.1
De facto but living apart	2.9	3.3	2.9–6.4
Unknown	10.9		
Official status (N = 105 cases)			
Married	35.0	45.7	36.2–55.2
Divorced^d^^, see also [^^26^^]^	15.3	20.0	15.3–27.7
No official status	26.3	34.3	26.3–43.4
Unknown	23.4		
Break-up of the relationship (N = 117 cases)			
No break-up reported	36.5	42.7	36.5–51.1
Yes, break-up within a month prior to the homicide	19.0	22.2	19.0–29.8
Yes, break-up > a month prior to the homicide^e^	29.9	35.0	29.9–43.7
Unknown	14.6		
Previous partner violence (N = 88 cases)			
Probably not	16.7	26,2	17.0–35.3
Probably yes	8.8	13.6	8.8–35.3
Certainly yes	38.7	60.2	50.0 = 70.5
Unknown	35.8		

^a^ 95% CI’s were adjusted when a boundary was impossible (see Table 1).

^b^ No other combination occurred for > 5% of the sample (valid %).

^c^ Some families were larger in reality, e.g. due to non-biological children.

^d^ In the general population in the Netherlands in 2015, 9.6% of 20–65 year olds were divorced [26].

^e^ This could range from several weeks to several years prior to the homicide.

### Prior exposure

For a large group of children (83%) among those for whom data on prior exposure were available, the reports described likely or confirmed previous violence in the home or neglect of the child (see Table 1). Considering only the most robust data, of the 116 children for whom previous exposure was confirmed, 43% had not received social or mental health care services before the homicide, while 41% of the exposed children had been in contact with these services, and for 16% it was unclear.

### Exposure to the homicide

Table 3 shows details of the homicide exposure of the children. For 80% of the children, the homicide occurred in their home environment, suggesting that their home became inaccessible soon afterwards, being a crime scene. For 36% of the children it was confirmed that they witnessed either the homicide or its consequences (e.g. the dead body). Applying the same counting method as Lewandowski and colleagues [7], in 43% of the families at least one child had witnessed the homicide or the crime scene, with a 95% confidence interval ranging from 34% to 52%. For 22% of the children, it was known that they were at the location of the homicide, but it was unclear to what extent they had heard or seen the event.

**Table 3:** Homicide exposure of the children (N = 256).

	%	Valid %	95% confidence interval for valid %^a^
Homicide in (one of) the home(s) of the child (N = 238)			
No	18.4	19.7	18.4–24.8
Yes	74.6	80.3	75.2–81.6
Unknown	7.0		
Location of the child (N = 213)			
Certainly not on location	34.4	41.3^b^	34.7–47.9^b^
Certainly on location, witness status unclear	18.4	22.1	18.4–27.6
Certainly on location, saw at least consequences	6.6	8.0	6.6–11.6
Certainly on location and direct witness	23.0	27.7	23.0–33.7
Direct witness and accomplice Unknown	0.8 16.8	0.9	0.8–2.2
Weapon/means of homicide (N = 245)			
Cutting weapon	49.2	51.4	49.2–53.5
Firearm	19.1	20.0	19.1–23.4
Strangulation	14.5	15.1	14.5–18.8
Other	12.9	13.5	12.9–17.2
Unknown	4.3		
Child physically harmed due to being attacked (N = 205)			
No	78.1	97.6	95.5–98.0
Yes	2.0	2.4	2.0–4.6
Unknown	19.9		
Suicide of perpetrator within 24hrs (N = 251)			
No	88.7	90.4	88.7–90.6
Yes	9.4	9.6	9.4–11.3
Unknown	2.0		

^a^95% CI’s were adjusted when a boundary was impossible (see Table 1)

^b^ 58.7% were ‘on location’ (95% CI 52.1–65.3).

Half of the children lost their parent by means of a cutting weapon. Firearms were the second most prevalent weapon used, whereas strangulation and other means were less frequently reported. Considering only those children who were possibly or certainly confronted with the crime scene, 83% had lost their parent by means of a cutting weapon or firearm. For a minority of the children, the perpetrator committed suicide within 24 hours after the homicide (10%, see Table 3).

## Discussion

Despite the global recognition of their importance, data on intimate partner homicide are hard to obtain, hampering human rights and public health efforts [6] [19]. Even less information is available regarding children bereaved by intimate partner homicide. Considering that more than 1 in 3 female homicides are due to intimate partner violence and that many of these women have children [6] [7], an understanding of children’s circumstances is essential to provide adequate trauma-informed care. We cross-examined 8 national data sources in the Netherlands and identified the full population of children bereaved by intimate partner homicide in the period 2003–2012. We examined their background and circumstances, both prior to and during the killing.

A key finding is that while a large number of children had been exposed to prior violence or neglect at home, this exposure was frequently discovered only after the homicide. Many of the exposed children had not received professional support regarding their experiences. While clinical case series indicate that children with prior exposure to violence have more psychological and behavioral difficulties than children who did not have this exposure [e.g. 10, 12], our findings suggest that this vulnerability is to be expected in the majority of children seen after a parental homicide. Domestic violence, child maltreatment, and child neglect are vulnerability factors for children’s development in many spheres of life, including mental health, social functioning, and academic performance [27–30]. Sustained and multiple exposure to violence during childhood is also a risk factor for violence perpetration later in life [16–18]. Not only direct paths appear to be relevant, but also indirect paths. For example, domestic violence is related to higher rates of depression among women, which affects their parenting capacity; a reality that several of the bereaved children may have encountered [31] [32]. The homicide suddenly adds grief and a violent disruption of daily life to this existing vulnerability.

Second, at least a third of the children witnessed the homicide or saw the crime scene. Considering that many of these killings took place with a cutting weapon or firearm, the children were likely exposed to shocking scenes, involving many graphic and olfactory cues. The proportion we found was lower than the proportion reported by Lewandowski and colleagues [7]; they reported children in 72% of the families saw the killing or the crime scene, while the upper boundary of our 95% confidence interval was 52%. Their sample appeared to have slightly younger children than our population, which may have meant that more children were with their mother at the time of the homicide, but this is unlikely to fully explain the difference in findings. Nevertheless, even our lower rates indicate that a substantial number of children are exposed to the homicide, suggesting that professionals involved shortly after fatal partner violence should enquire about children’s witness status and plan for addressing the exposure with effective interventions.

Moreover, clinically, we often hear family members and caregivers express the hope or assumption that the children have not really been exposed to the violence itself. However, Lewandowski’s and our reports suggest high exposure rates, and case studies have shown that even very young children can ‘take in’ and remember details of a murder, also when family members think that the children were too young or asleep [8],[33]. If the actual exposure is not properly assessed and non-exposure is assumed, incorrectly, an important opportunity to help a child process their experience is missed (this is not to suggest that children who were not witnesses may not be in need of mental health support). Trauma-focused treatments for children, such as trauma-focused cognitive behavioral therapy (TF-CBT) and eye-movement desensitization and reprocessing (EMDR) are effective interventions to address intrusive memories of the homicide [34],[35].

A third key finding was that immigrant children were overrepresented among children bereaved by intimate partner homicide compared children in the general population. About 60% of the children were first or second generation immigrants, for example from Morocco and Turkey. This finding is in line with an American study that found foreign country of birth to be a key risk factor for intimate partner femicide [36]. It is possible that immigrant status is a proxy for low socio-economic status; families living in poverty are at risk of various types of adversity including mental health problems and violence [7]. Irrespective of the cause of overrepresentation, our finding underscores the need to tailor services to the culture of the families involved. This may include the language in which the services are provided and extra outreach to ensure access to care [37].

Two further findings merit attention. First, we found that in many cases, a child’s home became a crime scene. A home sealed as a crime scene often means that a child no longer has access to personal items, including ones that could provide comfort such as favorite toys or belongings of the deceased parent [38]. We would suggest that professionals negotiate access to these items as soon as they are involved. Second, two out of three children were younger than 10 years old at the time of the homicide. Up to about 10 years of age, children are still developing an understanding of the concept of death as well as vocabulary to express complex and mixed feelings [39] [40]. This implies that many children may need extra support to understand what death means and how to express their experiences.

Even though determining risk factors for intimate partner homicide was not the aim of this study and we only considered victims who had children, our findings are broadly in line with previous international research. Studies in the USA and Europe [20],[35],[41–43] have singled out risk factors such as female gender, previous domestic violence, immigration status, and (recent) separation or divorce, all of which appeared to be overrepresented in our sample. However, with the exception of immigrant status, we have not formally tested this overrepresentation and no robust conclusions can be drawn.

Several practical implications of the current study have already been mentioned. In particular, our findings suggest that care after an intimate partner homicide needs to address a) previous (and potentially sustained) neglect, maltreatment and domestic violence experiences; b) exposure to the homicide and the crime scene; c) traumatic grief and bereavement; d) cultural differences, including possible language barriers; and e) severe disruption of daily life and its developmental implications. Many of these elements are also likely to be relevant to children who are exposed to near-fatal domestic violence. While it is impossible and unnecessary for all professionals within (mental) health care and child protection systems to be competent in all the areas mentioned, every system should be able to make the expertise available at short notice. Fatal domestic violence cases are often ‘high stakes’ for many reasons apart from the direct impact on the victim and the children. The impact on family members and the community as well as the extensive—and sometimes disruptive—media attention they receive make adequate care particularly challenging.

### Limitations and future directions

To our knowledge, this is the first study to obtain robust data on the full population of children affected by parental intimate partner homicide in a country over an extended period of time. The scarcely available previous studies were all clinical case studies or series, or depended on tracing, reaching and obtaining approval of family members, potentially introducing biases in findings [8]. While unique in its comprehensiveness, our study only involves one, high-income country; future studies should examine similarities and differences across countries with different legal, social and economic backgrounds. In addition, because we focused on pre- and peri-homicide factors that were reliably reported for as many children as possible, our analysis did not include mid- or long-term outcomes of children and their caregivers post-homicide. Previous reports have drawn attention to the impact of, for example, custody battles and the separation of siblings on children’s mental health, as well as to the burden on caregivers (e.g. [38],[44],[45]). These merit attention in further research. Another limitation concerns the exact information we could extract. Because of the variation in reporting and the lack of standardization, we were limited in the level of nuance that we could achieve. For example, the reports did not allow for separate coding of prior neglect and maltreatment. Also, while we assessed the information regarding prior services as relatively reliable, there may have been cases of underreporting. In addition, data regarding the socio-economic status of the families would have been informative. Overall, further standardization of reports of children’s demographics and history would be valuable for improving our understanding of the experiences and characteristics with which children are likely to present to services.

Future research should ideally be outcome-oriented, international, and working towards a predictive model. First, it will be important to collect data regarding children’s long-term mental health, development and behavior outcomes. Ideally, like the current study, this would not depend on convenience or clinical samples, but have its roots in population data. Second, future studies should combine data from multiple countries to allow for more advanced analyses and cross-country comparisons, similar to the efforts to improve data collection regarding femicides [46]. Third, when the first two items are addressed, it would be valuable to build a predictive model and test which factors are most important for child outcomes. Such a model could incorporate the recently proposed framework for relevant pre-, peri-, and post-homicide factors [8]. This endeavor needs not only longitudinal and robust data but also a substantial sample size. The ultimate outcome would be evidence-informed guidelines regarding key issues such as elements of effective support in the immediate aftermath, living arrangements and caregiver assistance, contact with the perpetrating parent, and treatment.
